# The frequency of dyslipidemia in patients with idiopathic tinnitus

**DOI:** 10.14744/nci.2021.90187

**Published:** 2022-07-06

**Authors:** Suleyman Erdogdu

**Affiliations:** Department of Otorhinolaryngology, University of Health Sciences Turkey, Haydarpasa Numune Training and Research Hospital, Istanbul, Turkiye

**Keywords:** Dyslipidemia, hypercholesterolemia, lipoproteins, tinnitus, triglycerides

## Abstract

**OBJECTIVE:**

We aimed to investigate the presence of dyslipidemia in blood biochemistry of patients with idiopathic tinnitus and to point out that dyslipidemia poses a risk to coronary artery disease.

**METHODS:**

Blood lipoprotein values and age and gender were compared between 158 patients with idiopathic tinnitus and 160 patients without tinnitus. Results were statistically evaluated.

**RESULTS:**

In total, more than half of 318 patients had high blood cholesterol levels. In both groups, the cholesterol average was 215 mg/dl. Furthermore, low density lipoprotein (LDL) and high density lipoprotein (HDL) levels were higher than normal. However, no statistically significant difference was determined in the comparison of Cholesterol, HDL, and LDL levels in both groups (p<0.05). However, serum triglyceride levels of patients with tinnitus were when compared with the control group, a statistically significant difference was found (p=0.001); The numbers of men and women were approximately close to each other and the number of patients between the ages of 50 and 60 was higher. The average age is 53. As a result, the risk of life-threatening coronary heart disease increases, as the majority of patients with tinnitus are in the middle age group and have high lipoprotein values.

**CONCLUSION:**

In patients presenting with the complaint of tinnitus, high serum lipoprotein values may not be directly related to tinnitus. However, it should be remembered that the presence of dyslipidemia may affect coronary vessels and lead to coronary artery diseases. However, a significant correlation was found between high serum triglyceride levels and tinnitus. In addition, if the patient has dyslipidemia, it will be healthy to treat dyslipidemia.

## Highlight key points

- The most frequent form of dyslipidemia is hyperlipidemia.
- In patients with idiopathic tinnitus,blood lipoprotein values were found to be high
- The relationship between high serum triglyceride levels and tinnitus was found to be significant.

Approximately 10% of the population suffer from tinnitus. Tinnitus is a common and disturbing symptom characterized by perception of sound with the absence of external stimuli. There are differences in etiology in patients with tinnitus. Tinnitus reaction varies from simple to severe irritation. Some people have hearing difficulty due to tinnitus. Severe tinnitus causes many psychological symptoms such as nervousness, frustration, impaired concentration, and sleep disturbance [1].

Hyperlipidemias are the most common type of dyslipidemias. These are also called hyperlipoproteinemias, which blood lipid levels are high. High low density lipoprotein (LDL) has clearly played a role in atherosclerosis and coronary artery disease. Conversely, high density lipoprotein (HDL) levels are often preferable and can reduce the risk of coronary artery disease. Triglyceride levels are high in humans due to the recent diet of fatty foods [2].

Many researchers found high triglyceride, high cholesterol, high LDL, and low HDL in the serum of patients with tinnitus. But this high was not statistically significant [3–5].

## MATERIALS AND METHODS

Patients with tinnitus complaints were included in the study. Patients with tinnitus, hearing loss, head trauma, otologic disease, and objective tinnitus were excluded from this study. Patients with only idiopathic tinnitus were included in this study. 158 patients whose blood lipoprotein levels were measured were included in the study. Furthermore, 160 patients without tinnitus and whose blood lipoprotein levels were checked were included in the study as a control group. The blood lipoprotein values of a total of 318 patients were compared with their age and gender, and the results were evaluated statistically.

As lipid profiles of these patients are evaluated in blood samples: Triglycerides levels of <200 mg/dl were regarded as average, borderline 200–400 mg/dl was moderate, border lining 400–1000 mg/dl was high, and ultimately 1000 mg/dl was considered to be very strong; values of cholesterol of <200 mg/dl were considered normal. LDL values below 130 mg/dl, 130–159 mg/dl and <160 mg/dl are considered desirable; amounts of HDL <40 mg/dl are considered low, and 40 mg/dl are considered desirable in accordance with the standards defined by American Heart Association [6].

### Statistical Analysis

Statistical Package for the Social Sciences 15.00 package program was used frequency distribution in descriptive statistics of data average. Standard deviation and t-test were used.

### Ethics Committee Approval

The Ethics Committee of University of Health Sciences Haydarpasa Numune Training and Research Hospital provided the ethics committee approval for this study (number: 2021/3309).

## RESULTS

As a result of examining the retrospective records of 318 patients between January 2012 and January 2020, serum cholesterol, LDL, and HDL levels were found to be higher than normal in most cases, while triglyceride levels were lower than normal in most cases (Table 1).

**Table 1:** Age and gender distributions of cases

	Patient group (n=158) %	Control group (n=160) %
Age		
≤29	6.3	6.9
30–39	13.3	13.1
40–49	18.4	17.5
50–59	27.2	26.9
60–69	19.6	20.6
≥70	15.2	15.0
Gender		
Female	53.2	50.6
Male	46.8	49.4

The female and male numbers of the cases are approximately equal and the number of patients between the ages of 50–60 is higher. The average age of both groups is 53 (Table 2).

**Table 2:** Average and standard deviation results of the patient’s findings

	Patient group Mean±SD	Control group Mean±SD	p
Age	52.77±14.91	52.89±15.79	0.944
Cholesterol	215.53±44.15	214.34±48.88	0.819
HDL	50.85±15.52	46.69±13.09	0.010*
LDL	138.84±43.04	145.28±64.81	0.298
Triglyceride	136.68±66.99	172.64±107.10	0.001*

* : P<0.05; HDL: High density lipoprotein; LDL: Low density lipoprotein; SD: Standard deviation.

In the data of 318 patients, the cholesterol average was 215 mg/dl in both groups. HDL mean was 50.85±15.52 mg/dl in tinnitus and 46.69±13.09 mg/dl in the control group; The mean LDL was 138.84±43.04 mg/dl in tinnitus and 145.28±64.81 mg/dl in the control patients; triglyceride mean was 136.68±66.99 mg/dl in tinnitus and 172.64±107.10 mg/dl in the control group (Table 3).

**Table 3:** Serum lipoprotein levels of patients

	Patient group (n=158)		Control group (n=160)	
	Female %	Male %	Female %	Male %
Cholesterol				
<200 mg/dl	21.52	13.92	15.62	25.00
200–239 mg/dl	14.56	21.52	13.12	16.88
≥240 mg/dl	17.09	11.39	20.63	8.75
HDL				
<40 mg/dl	5.70	18.98	8.75	23.75
≥40 mg/dl	47.47	27.85	40.62	26.88
LDL				
<130 mg/dl	24.68	20.89	19.37	24.38
130–159 mg/dl	12.66	13.29	13.12	13.75
≥160 mg/dl	15.82	12.66	16.88	12.50
Triglyceride				
<200 mg/dl	46.83	36.71	35.62	33.75
200–400 mg/dl	6.33	10.13	13.75	16.88

HDL: High density lipoprotein; LDL: Low density lipoprotein.

When the age, cholesterol, and LDL values of the male and female patients with tinnitus and the female and male patients in the control group were examined, no statistically significant difference was found (p>0.05). When the patients and control groups were compared, serum HDL level was statistically significant (p=0.010). A statistically significant difference was found between the triglyceride values of female and male patients with tinnitus and female and male patients in the control group (p<0.005).

## DISCUSSION

Tinnitus is an imaginary auditory perception that occurs in humans. Tinnitus is an imaginary auditory perception that occurs in some people. Tinnitus is a disturbing problem that affects many people around the world and it is perceived as ringing in the ears. There is no effective drug treatment. However, much research continues on the treatment and its mechanism. The definitive treatment, physiopathology, and etiology of tinnitus are not yet mature. Before the treatment, appropriate clinical evaluation with a detailed history, measurement of the amount of hearing loss, measurement of tinnitus severity and determination of symptoms, and comorbidities related to etiological factors should be done. Successful treatment of tinnitus depends on teamwork consisting of otolaryngologist, audiologist, neurologists, psychologists, sleep, and pain experts [7]. Tinnitus is a complex symptom that requires a comprehensive multidisciplinary evaluation [8]. Tinnitus presents in 10–15% of the population. Treatment of patients with refractory tinnitus may be inadequate, then the presence of tinnitus reduces the patient’s life quality, so patients may seek a different doctor [9].

Most of the patients with tinnitus are chronic. Development of molecular, biochemical and imaging techniques have helped new researches for causes and treatment of tinnitus. Tinnitus can originate anywhere along the auditory pathways from the cochlear nucleus to the auditory cortex. There are theories that it is mainly caused by injured cochlear haircells [10].

The effects of technological developments are very important in the advancement of research on tinnitus. For example, neuroimaging with functional magnetic resonance imaging and positron emission tomography provides important data for pathophysiology-oriented studies. Technological innovations have been useful in diagnosing objective tinnitus [11].

Tinnitus is a common problem and adversely affects the quality of life. The prevalence of tinnitus in the USA is one of ten adults. The duration of exposure to noise and tinnitus are proportional and both are possible risk factors. It is more common in those who are regularly exposed to noisy environments at work and during their leisure time [12]. Education and rehabilitation should be done to minimize the negative effects of tinnitus [13].

Tinnitus is frequently observed not only in those with sleep disorders, but also those without sleep problems have tinnitus. On the other hand, the severity of insomnia is not related to the duration of tinnitus or age. Chronic tinnitus patients have sleep difficulties [14].

Plasma lipoproteins transport lipids to tissues for energy use, lipid storage, steroid hormone production and bile acid formation. Lipoproteins consist of free and bound cholesterol, triglycerides, phospholipids, and protein compounds called dapolipoproteins that have effects such as enzyme activation and inhibition, molecules, and structural elements that bind to cellular receptors [15].

Cholesterol has effects particularly on atherosclerosis and coronary artery disease. Total cholesterol is recognized by three lipoprotein fractions: HDL, LDL, and very low density lipoproteins. Lipoproteins are spherical macromolecule complexes made up of lipids. These lipids consist of free and bound cholesterol, triglycerides, and phospholipids. Proteins called apoproteins provide structural stability to cholesterol and also play a critical role in determining the metabolic fate of particles [2]. Patients with tinnitus should also be evaluated in this respect.

Etemadi et al. [3], in their study on 76 patients, found that patients with tinnitus with dyslipidemia were the most with hypercholesterolemia (23.68%), and in the second place high serum triglyceride (11.84%) and low HDL (9.21%) and high LDL (5.26%), respectively. They stated that the prevalence of dyslipidemia in patients with tinnitus is not statistically significant when compared with the general population. Many researchers have detected high and it is not statistically significant (p>0.05).

Lee et al. [4] showed that there was little change in blood biochemistry results in elderly people with hearing measurements performed in 217 volunteers between the ages of 60–82 and that the total cholesterol and LDL levels were slightly higher than normal, but were not statistically significant.

In this study, a statistically significant difference was found between the triglyceride values of female and male patients with tinnitus and female and male patients in the control group (p<0.05). The HDL level results in the serum of the patients and the control group were normal. In fact, it is desirable to have a high HDL level for a healthy life. Low HDL levels are not considered healthy.

Pulec et al. [5] found the frequency of hypercholesterolemia in tinnitus patients to be 5.1%. In their studies, they showed that the hypolipidemic diet improved the serum lipid profile as well as tinnitus. The researchers noted that in the case of hypercholesterolemia, the problem in the inner ear is chronic obstruction of the capillary portion of the stiff vessels, which can lead to both a biochemical change in the endolymphatic area and ischemia. They explained that tinnitus can be improved with a hypolipidemic diet.

Rosen [16] showed in his epidemiological study that there was a high link between coronary artery disease, atherosclerosis, and high cholesterol in people fed saturated fats and hearing loss.

Very high triglyceride levels create the risk of pancreatitis. Very low total cholesterol levels are the other part of dyslipidemias, but they are rare. Low cholesterol, cancer, liver cirrhosis may be related to respiratory problems and acute disease [2].

Hyperlipoproteinemia is a common risk factor that increases coronary artery disease with atherosclerosis it causes. In addition, the risk increases more with hypertension, Type 2 diabetes mellitus, high body mass ındex, age and gender, and the presence of smoking factors [17].

It is necessary to treat the dyslipidemia of patients with tinnitus complaints, considering that it may progress.

Hyperlipidemia is an inherited high risk factor for coronary artery disease, a directly modifiable risk factor. Therefore, the risk of life-threatening coronary heart disease increases, since the majority of patients with tinnitus are in the middle age group and have high lipoprotein values [18]. The hyperlipidemia of tinnitus patients should be treated, as these researchers noted. There is no gold standard in the treatment of tinnitus [19].

There is no particularly recommended drug for treatment. There are few treatment options and available drugs aim to reduce the effect of tinnitus [20] Variability in tinnitus treatment results may be due to differences in individual patients’ characteristics [8].

## Conclusion

In patients with idiopathic tinnitus, blood lipoprotein values were found to be high as in other studies in the literature. In this study, the relationship between high serum triglyceride levels and tinnitus was found to be significant. Therefore, considering the complications of dyslipidemia, these patients need medication and diet therapy to normalize their blood lipoprotein values. There is a need for a multidisciplinary approach. If the patient’s blood biochemistry results are impaired, treating the patient’s dyslipidemia will cause healthy living.
